# Dynamic aortic regurgitation caused by suction-induced right coronary cusp entrapment in a ruptured right sinus of Valsalva aneurysm: a case report

**DOI:** 10.1093/ehjcr/ytag149

**Published:** 2026-03-10

**Authors:** Qingyan Ma, Ying Zhang

**Affiliations:** Department of Cardiovascular Function Diagnostics, Guangdong Provincial People's Hospital (Guangdong Academy of Medical Sciences), Southern Medical University, No. 106, Zhongshan Second Road, Guangzhou 510080, China; Department of Cardiology, Guangdong Provincial People's Hospital (Guangdong Academy of Medical Sciences), Southern Medical University, No. 106, Zhongshan Second Road, Guangzhou 510080, China

**Keywords:** Aortic regurgitation, Sinus of Valsalva aneurysm, Cusp entrapment, Ventricular premature contractions, Multimodality imaging, Valve-sparing repair, Case report

## Abstract

**Background:**

Aortic regurgitation (AR) is usually caused by intrinsic cusp pathology or aortic root dilatation. Beat-to-beat variability in AR severity, sometimes accentuated by rhythm disturbances, is uncommon and may be overlooked without rhythm-aware, multimodality imaging across multiple cardiac cycles.

**Case summary:**

A 30-year-old woman with prior ventricular septal defect repair presented with recurrent palpitations and frequent ventricular premature contractions (PVCs). Transthoracic echocardiography identified a ruptured right sinus of Valsalva aneurysm with intermittent severe AR, more pronounced during premature beats. Three-dimensional transoesophageal echocardiography demonstrated a structurally intact right coronary cusp that was intermittently drawn into the rupture orifice during diastole, consistent with a suction-mediated functional mechanism producing beat-to-beat variation in regurgitation severity. Cardiac computed tomography confirmed rupture morphology and excluded aortic root dilatation. Valve-sparing surgical repair abolished cusp entrapment and restored leaflet coaptation, with mild residual AR on intraoperative assessment. At 2-year follow-up, transthoracic echocardiography showed stable mild AR without progression, and the patient remained asymptomatic.

**Discussion:**

This case illustrates a rare functional mechanism of dynamic AR caused by suction-induced cusp entrapment in a ruptured sinus of Valsalva aneurysm and underscores the value of rhythm-aware, stepwise multimodality imaging to guide valve-sparing management when cusp morphology is preserved.

Learning points

- Ruptured sinus of Valsalva aneurysm can produce dynamic, intermittent aortic regurgitation through a functional cusp entrapment mechanism.
- When regurgitation severity varies beat-to-beat, rhythm-aware multimodality imaging across multiple cycles is essential to avoid misclassification of the AR mechanism.
- Valve-sparing repair is feasible when cusp morphology is preserved and regurgitation arises from reversible functional distortion rather than intrinsic leaflet disease.

## Introduction

Dynamic or rhythm-modulated aortic regurgitation (AR) is uncommon and may be overlooked unless imaging is assessed across multiple cardiac cycles with attention to rhythm. We report a case of intermittent severe AR caused by suction-induced entrapment of the right coronary cusp at the rupture orifice of a ruptured right sinus of Valsalva aneurysm. A stepwise workflow (TTE → 3D-TEE → CT) clarified the functional mechanism in the setting of preserved cusp integrity and supported a valve-sparing repair strategy. Although ruptured sinus of Valsalva aneurysm has been reported previously,^1,2^ detailed multimodality demonstration of suction-driven intermittent cusp entrapment with preserved cusp morphology remains rare.

## Summary figure

**Figure:**
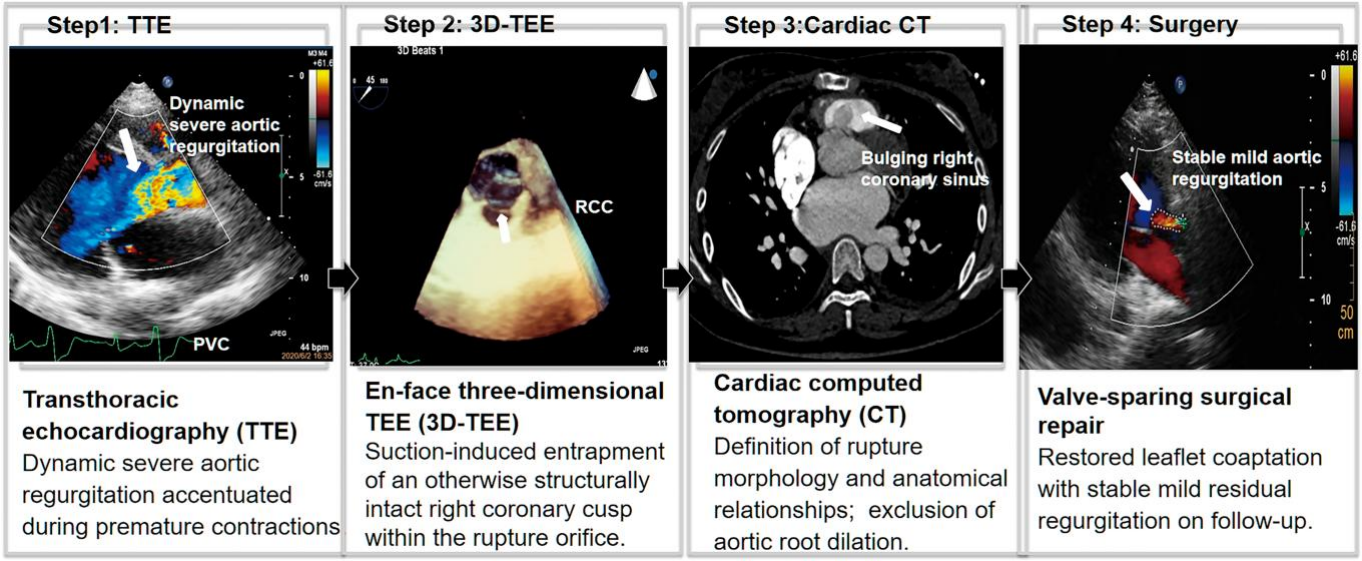
Stepwise multimodality diagnostic approach in a patient with ruptured right sinus of Valsalva aneurysm and dynamic AR. Transthoracic echocardiography identified intermittent severe regurgitation; three-dimensional transoesophageal echocardiography demonstrated suction-induced entrapment of a structurally intact right coronary cusp; and cardiac computed tomography provided complementary anatomical definition of rupture morphology and relationships. This integrated imaging strategy supported valve-sparing surgical repair with durable clinical improvement. The schematic illustration complements the clinical images and facilitates understanding of the proposed mechanism.

## Case presentation

A 30-year-old woman with a history of surgical repair of a ventricular septal defect presented with recurrent palpitations. A continuous murmur was heard along the left sternal border. Electrocardiogram demonstrated frequent ventricular premature contractions (PVCs), and NT-proBNP was elevated (2494 pg/mL). On admission, blood pressure was 135/45 mmHg, and she had no prior history of hypertension. During hospitalization, repeated non-invasive measurements ranged from 110–135 to 45–66 mmHg.

**Figure 1:**
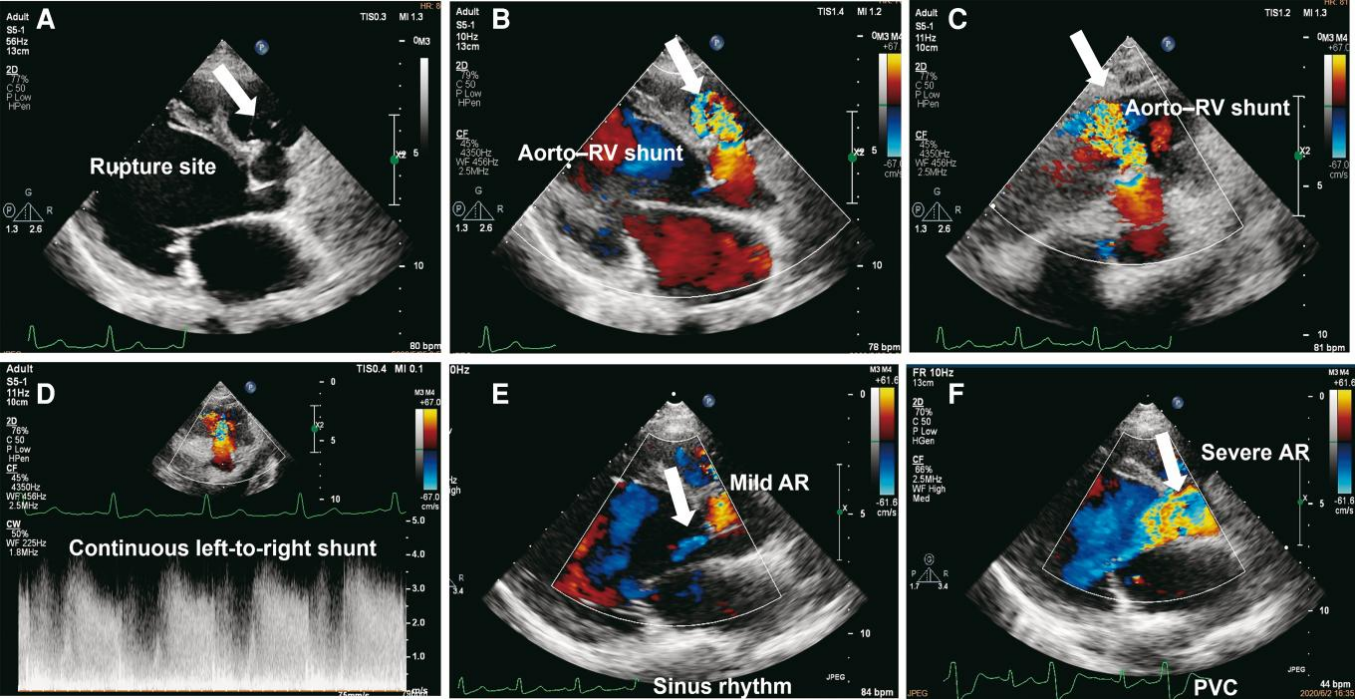
Transthoracic echocardiography (TTE) shows a ruptured right sinus of Valsalva aneurysm (RSVA) protruding into the right ventricle with continuous left-to-right shunting (*A–D*). In the same colour Doppler plane, aortic regurgitation is mild during sinus rhythm (*E*) but becomes severe during a premature ventricular contraction (*F*).

**Figure 2:**
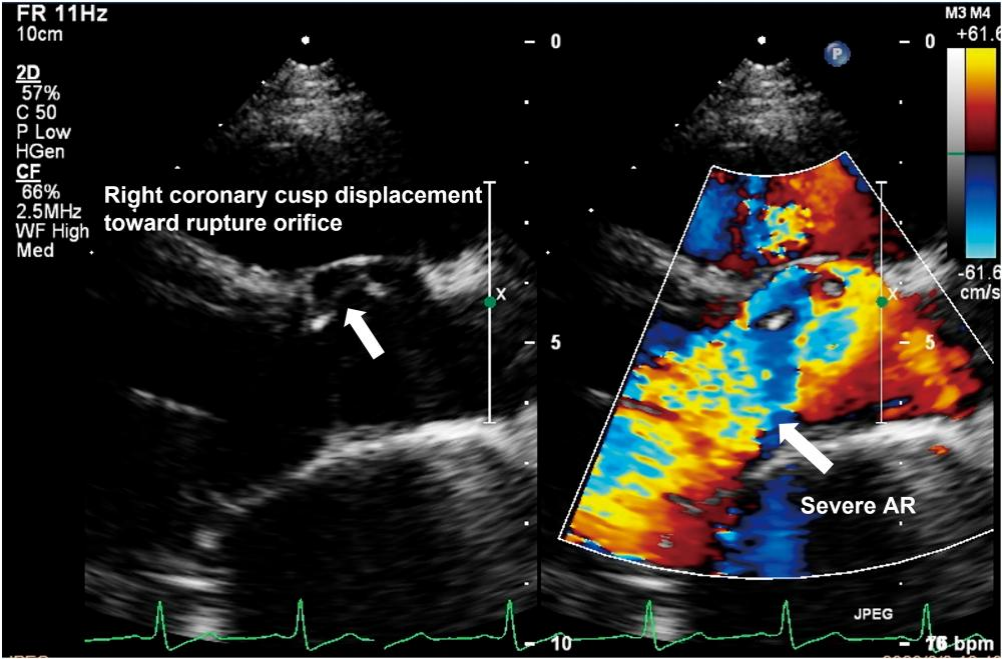
Parasternal long-axis TTE. (*A*) Diastolic malcoaptation with the right coronary cusp held open (white arrow). (*B*) Colour Doppler shows severe aortic regurgitation (white arrow).

Transthoracic echocardiography (TTE) revealed a ruptured right sinus of Valsalva aneurysm (RSVA) protruding into the right ventricle with continuous left-to-right shunting (*Figure 1A–D*). Intermittent severe AR was observed, with marked augmentation during PVCs (*Figure 1E* and *F*; Supplementary material online, *Videos S1* and *S2*). Magnified TTE suggested diastolic displacement of the right coronary cusp towards the rupture orifice (*Figure 2*; Supplementary material online, *Video S3*).

To determine whether the fluctuation in AR severity was rhythm-dependent or structurally mediated, β-blocker therapy was initiated to suppress PVCs. Metoprolol tartrate (50 mg twice daily) was started. Although 24-h Holter monitoring was not performed, continuous inpatient telemetry allowed an approximate, telemetry-derived (extrapolated) estimation of ectopic burden. Prior to β-blocker therapy, ventricular ectopy was estimated at ∼30%–40% of total beats over 24 h; after therapy, the estimated ectopic burden decreased to ∼5%–10%. Despite substantial suppression of ventricular ectopy, intermittent severe AR persisted.

**Figure 3:**
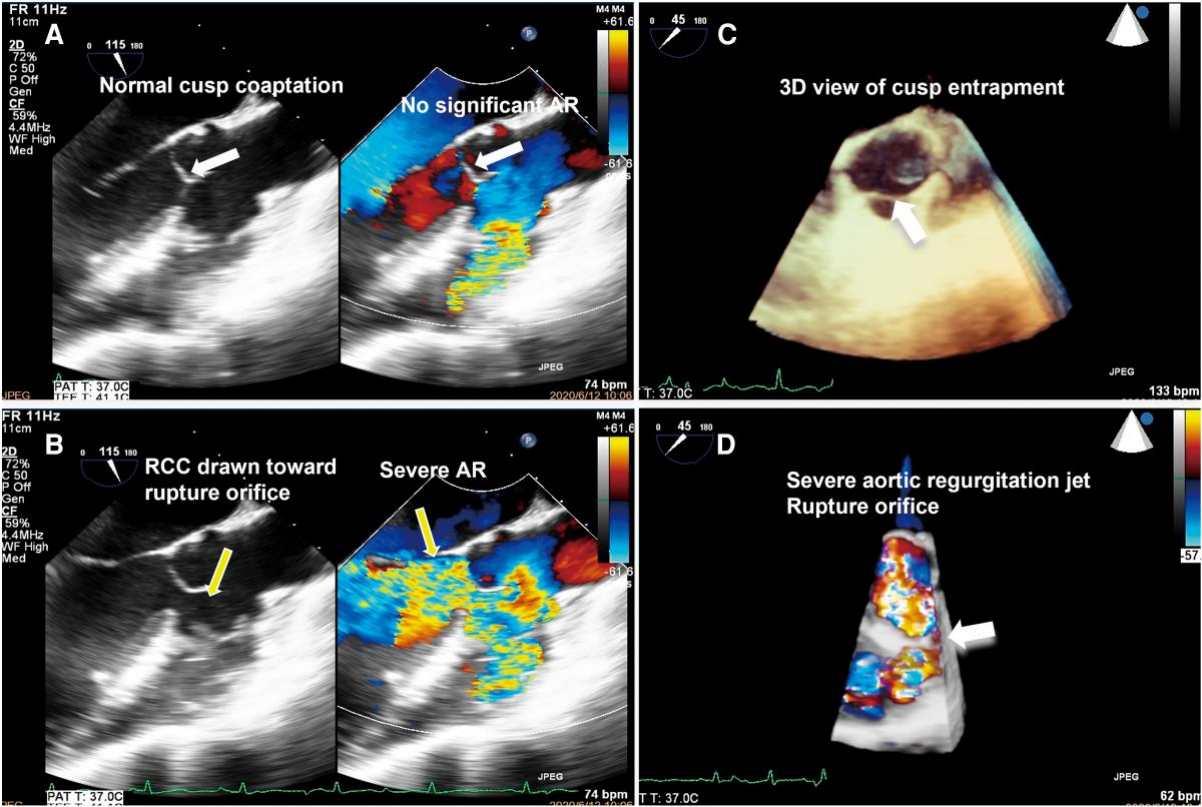
Three-dimensional transoesophageal echocardiography (3D-TEE) demonstrating intermittent severe aortic regurgitation caused by dynamic right coronary cusp entrapment at the rupture orifice. (*A*) Normal aortic valve closure without significant regurgitation (white arrow). (*B*) Suction-induced diastolic opening of the right coronary cusp with severe aortic regurgitation (yellow arrow). (*C* and *D*) Three-dimensional rendered views show intermittent entrapment of the right coronary cusp by the rupture defect, resulting in severe aortic regurgitation.

Three-dimensional transoesophageal echocardiography (3D-TEE) provided en face visualization of the aortic valve. All three cusps were structurally intact, but the right coronary cusp was intermittently drawn into the rupture orifice by a suction effect during diastole, producing beat-to-beat variation in AR severity independent of rhythm (*Figure 3*; Supplementary material online, *Videos S4* and *S5*).

**Figure 4:**
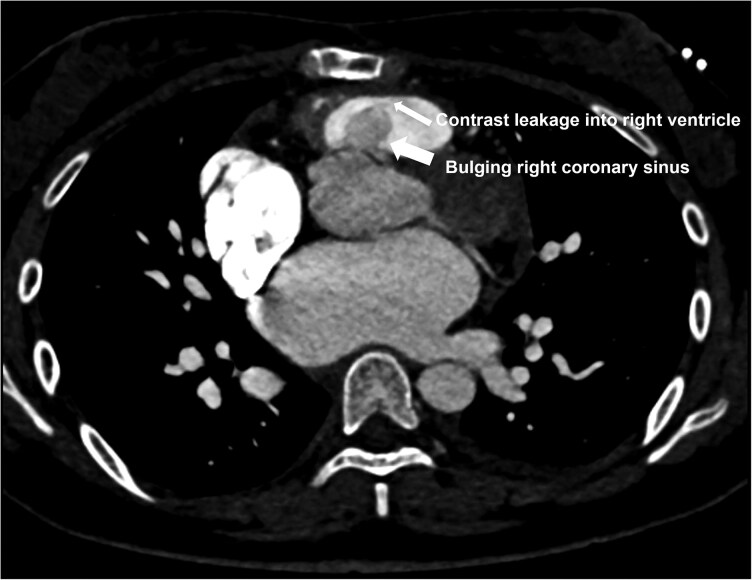
Cardiac computed tomography angiography (CTA) demonstrating rupture morphology. CTA shows bulging of the right coronary sinus towards the right ventricular outflow tract with contrast leakage into the right ventricle (white arrows), consistent with a ruptured right sinus of Valsalva aneurysm.

Cardiac CT angiography further delineated the ruptured aneurysm and excluded aortic root dilatation, confirming a functional rather than intrinsic leaflet mechanism (*Figure 4*).

**Figure 5:**
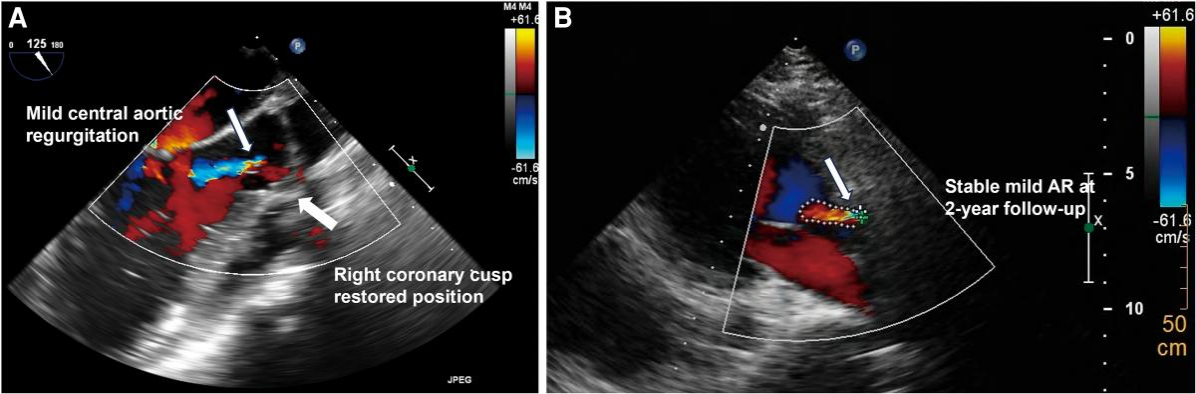
Post-repair and follow-up echocardiographic assessment. (*A*) Intraoperative transoesophageal echocardiography immediately after valve-sparing repair demonstrating restored cusp coaptation with mild residual central aortic regurgitation. (*B*) Transthoracic echocardiography at 2-year follow-up showing stable mild aortic regurgitation without progression.

A multidisciplinary heart team recommended valve-sparing repair. Following completion of multimodality imaging and heart team discussion, valve-sparing repair was performed 19 days after admission. The patient underwent surgical closure of the rupture and aortic valve plasty. Intraoperative TEE demonstrated restored leaflet coaptation with mild central AR (*Figure 5A*). At 2-year follow-up, TTE showed stable mild AR without progression (*Figure 5B*), and the patient remained asymptomatic.

## Discussion

Although AR is most commonly caused by intrinsic leaflet disease or aortic root dilatation, dynamic or functional mechanisms are less common and can be challenging to diagnose because regurgitant severity may vary with loading conditions and rhythm.^3–5^ In this case, AR resulted from suction-induced entrapment of the right coronary cusp into the rupture orifice of a ruptured sinus of Valsalva aneurysm, producing intermittent beat-to-beat variability in regurgitation severity. A rhythm-aware, stepwise imaging approach was therefore critical.^4,6,7^ Transthoracic echocardiography identified the ruptured aneurysm and fluctuating AR, with worsening during premature beats. Despite marked suppression of premature beats after β-blocker therapy, intermittent severe AR persisted, suggesting that rhythm disturbance was not the sole driver of regurgitation variability and supporting a predominantly functional, anatomy-related mechanism. 3D-TEE was indispensable for confirming this mechanism,^4,6,7^ demonstrating preserved cusp morphology and dynamic suction-mediated cusp entrapment. Cardiac CT further provided complementary anatomic definition by delineating rupture morphology and excluding aortic root dilatation.^5,6^

The ruptured sinus of Valsalva aneurysm in this patient was considered most consistent with a congenital lesion in the context of a history of ventricular septal defect repair.^2^ There was no evidence of aneurysm rupture at the time of the prior repair, and the patient remained asymptomatic for many years thereafter, supporting late rupture of a pre-existing aneurysm rather than an iatrogenic or post-operative cause.

Mechanistic clarification directly informed treatment. Because cusp integrity was preserved and AR arose from reversible distortion rather than intrinsic leaflet degeneration, valve-sparing repair was favoured, consistent with guideline principles recommending repair when feasible and when leaflet structure is preserved.^8^ Surgical correction of the rupture eliminated the suction effect, with durable clinical improvement.

In summary, this case demonstrates a rare dynamic mechanism of AR caused by suction-induced right coronary cusp entrapment in a ruptured sinus of Valsalva aneurysm. Diagnosis required sequential, rhythm-aware multimodality imaging. Recognizing the functional mechanism enabled successful valve-sparing repair and durable clinical improvement. This case underscores the importance of assessing AR across multiple cardiac cycles and imaging planes when regurgitant severity fluctuates.

## Patient’s perspective

The patient reported relief of symptoms and satisfaction with preserving her native valve.

## Supplementary Material

ytag149_Supplementary_Data

## Data Availability

Data are available from the corresponding author upon reasonable request.

## Lead author biography

Qingyan Ma graduated from the Guangdong Cardiovascular Institute in 2014 and has been working as a cardiologist in the Department of Cardiovascular Function Diagnostics at Guangdong Provincial People's Hospital since 2014. She specialized in clinical and research imaging fields such as echocardiography and electrocardiography.
